# Chemical Synthesis and Biological Evaluation of Sulfated DK4 Derivatives from *Escherichia coli* K4 Polysaccharide

**DOI:** 10.3390/molecules31152721

**Published:** 2026-08-05

**Authors:** Yajia Wang, Yue Chen, Meiling Qiu, Shuqin Xu

**Affiliations:** 1 School of Life Science and Health Engineering, Jiangnan University, Wuxi 214122, China; wangyajia000@163.com (Y.W.); cy17773884210@163.com (Y.C.); 18812100209@163.com (M.Q.); 2 Key Laboratory of Carbohydrate Chemistry and Biotechnology, Ministry of Education, Jiangnan University, Wuxi 214122, China; 3 Centre for Misfolding Diseases, Yusuf Hamied Department of Chemistry, University of Cambridge, Cambridge CB2 1EW, UK

**Keywords:** defructosylated K4, NMR, sulfation, bioactivity

## Abstract

Three sulfated derivatives were synthesized using microbially derived defructosylated K4 (DK4) polysaccharide as a chondroitin-like polysaccharide backbone. The obtained derivatives were structurally characterized by nuclear magnetic resonance spectroscopy, Fourier-transform infrared spectroscopy, elemental analysis, and disaccharide composition analysis. The results demonstrated that sulfate groups were successfully introduced into the DK4 structure, while the main chondroitin-like polysaccharide backbone of DK4 was largely preserved after sulfation. Further biological evaluation indicated that the sulfated DK4 derivatives differentially altered the expression levels of Ras homolog family member A/Rho-associated coiled-coil-containing protein kinase 1 (RhoA/ROCK1) and myelin basic protein (MBP). This work provides an experimental basis for further elucidating the structure–activity relationship between sulfation patterns and glial cell functions.

## 1. Introduction

Chondroitin sulfate (CS) is a sulfated glycosaminoglycan (GAG) widely distributed in the extracellular matrix (ECM) and on cell surfaces [1]. In recent years, CS has attracted increasing attention because of its diverse biological activities involved in various physiological and pathological processes, including osteoarthritis [2], neural injury [3], and neurodegenerative diseases [4]. CS is a linear polysaccharide composed of repeating disaccharide units of glucuronic acid (GlcA) and N-acetylgalactosamine (GalNAc), and its structural heterogeneity primarily arises from variations in sulfation patterns [5,6,7,8]. Its biological functions are closely associated with its specific carbohydrate chain composition, degree of sulfation, and spatial conformation [9,10]. Based on the position of sulfate groups, CS can be classified into several subtypes, such as chondroitin sulfate A (CSA), chondroitin sulfate C (CSC), and chondroitin sulfate E (CSE). Different sulfation patterns result in distinct charge distributions and conformational features, which are considered important structural determinants affecting the biological activity of CS and its interactions with proteins [11]. Therefore, different sulfated sequences may confer distinct biological functions on CS.

In the nervous system, chondroitin sulfate proteoglycans can interact with receptors such as protein tyrosine phosphatase sigma (PTPσ) [12], leukocyte common antigen-related phosphatase (LAR) [13], and Nogo receptors [14], which are involved in the regulation of downstream signaling pathways, including the RhoA/ROCK pathway. CSE has been shown to exhibit stronger recognition by PTPσ than several other CS sulfation motifs, implying that densely sulfated CS domains may have a greater capacity to regulate PTPσ-associated signaling. In addition, CSPGs have been reported to suppress the behavior of neural precursor cells through LAR and RPTPσ, with concomitant involvement of the Rho/ROCK pathway, supporting a functional link between CS–receptor engagement and downstream cytoskeletal regulation. These findings suggest that structural differences among CSA, CSC, and CSE may produce distinct biological responses by altering their binding strength or interaction mode with PTPσ and related receptor proteins. Compared with monosulfated CSA and CSC, disulfated CSE exhibits stronger activity in promoting neurite outgrowth [15]. Further studies have shown that CSE tetrasaccharides can significantly increase the number of neurites in primary hippocampal neurons and promote the elongation of major neurites, whereas CSE disaccharides and unsulfated tetrasaccharides show no obvious effects [16]. These findings indicate that both the sulfation pattern and oligosaccharide length are critical determinants of this type of neurobiological activity. In addition to neurite outgrowth, oversulfated CS/dermatan sulfate (CS/DS) containing E units can interact with L-selectin, P-selectin, and various chemokines [17]. Moreover, structurally homogeneous CSE 19-mers have been reported to alleviate LPS-induced injury by targeting extracellular proteins, suggesting that highly sulfated CS may have potential advantages in inflammation-related molecular recognition and the regulation of inflammatory injury [18]. Therefore, under the premise of defined structure and biological safety, appropriately increasing the sulfation degree of CS derivatives may represent a reasonable strategy to expand the application of chondroitin sulfate derivatives in central nervous system repair and inflammatory regulation. Traditionally, CS is mainly isolated from animal cartilage, such as bovine, porcine, or shark cartilage; however, this approach has several limitations, including strong dependence on animal sources, significant batch-to-batch variation, and potential pathogen contamination [19,20].

In recent years, increasing efforts have been directed toward green synthetic strategies based on microbial fermentation and enzymatic catalysis. *Escherichia coli* O5:K4:H4 naturally produces a capsular polysaccharide, known as K4 polysaccharide, whose repeating disaccharide unit is highly similar to the backbone structure of chondroitin sulfate [21]. The capsular polysaccharide produced by *Escherichia coli* K4 contains a chondroitin-like GlcA-GalNAc backbone bearing fructose branches [22]. Therefore, K4 polysaccharide has been regarded as an important precursor for the synthesis of CS and its derivatives. Studies on K4 polysaccharide have mainly focused on expanding its application as a glycosaminoglycan precursor [23], metabolic engineering [24], and fermentation optimization [25]. Previous studies have successfully improved the production of K4 polysaccharide through engineered strains and obtained products structurally closer to natural CS by combining enzymatic or chemical defructosylation strategies [26]. In addition, complete microbial cell factories have been constructed for the one-step or multienzyme cascade synthesis of chondroitin sulfate and its sulfated derivatives [27]. Thus, defructosylated K4 polysaccharide provides an important platform for the controllable preparation of highly sulfated chondroitin derivatives by enabling precise regulation of sulfation sites through enzymatic or chemical modification [28].

CS is generally considered an inhibitory glycosaminoglycan in nerve regeneration, whereas heparan sulfate (HS) can act as a co-ligand to promote neurite outgrowth [29]. Although CS and HS share similar linear polysaccharide architectures, they exert distinct regulatory effects on neural responses, which may be closely associated with their disaccharide composition, sulfation degree, and sulfation pattern [11]. Previous studies have shown that monosulfated defructosylated K4 (DK4) derivatives inhibit cell viability and neurite extension, while dual-sulfated DK4 derivatives promote cell viability and increase neurite length, suggesting that the sulfation pattern of CS plays an important role in nerve regeneration-related processes [26]. Therefore, in this study, three sulfated derivatives were chemically synthesized from non-animal-derived DK4 polysaccharide. Their structural features and sulfation degrees were confirmed using multiple characterization methods. Based on previous cell-based studies of chondroitin sulfate and related sulfated polysaccharides, a 48 h exposure period was selected to evaluate the sustained cellular responses to the DK4 derivatives [30,31]. This endpoint maintained consistency with the subsequent protein-expression analyses. This study provides an experimental basis for the preparation of multiply sulfated CS derivatives and their potential application in regulating glial cell functions.

## 2. Results and Discussion

### 2.1. Chemical Characterization of DK4 Derivatives

**Figure 1:**
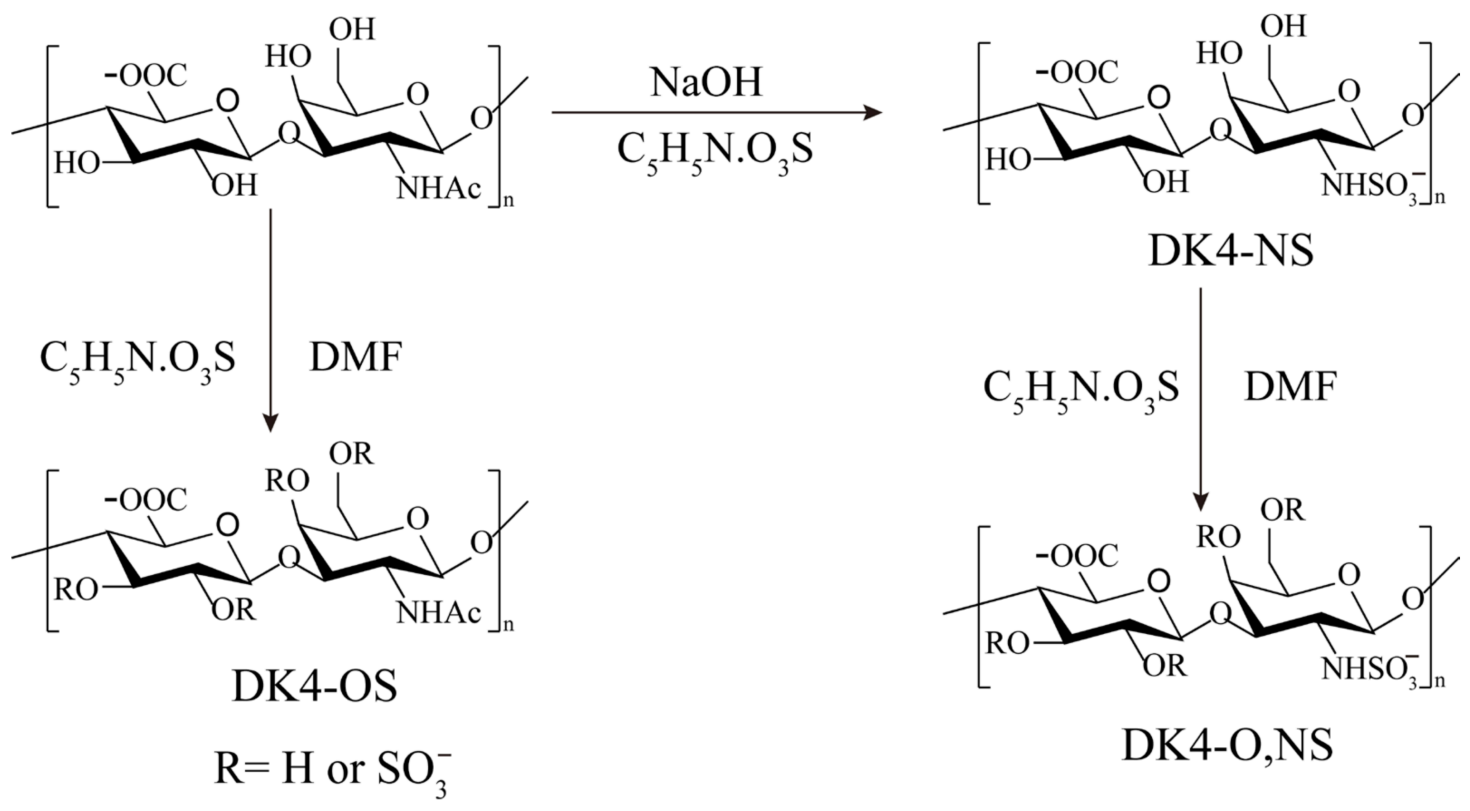
The synthetic route of sulfated DK4 derivatives (DK4-OS, DK4-O,NS, and DK4-NS).

This work provides a microbial-chemical strategy to produce three DK4 derivatives, including DK4-OS, DK4-O,NS, and DK4-NS. In our previous work, microbial-sourced K4 polysaccharide was obtained from engineered *Escherichia coli* K4 [32]. After removal of the fructose branches by mild acid hydrolysis, DK4 was obtained as a chondroitin analogue and subsequently used as the precursor for the synthesis of sulfated DK4 derivatives (Figure 1).

DK4 derivatives were chemically synthesized using DK4 as the polysaccharide backbone. In this study, the sulfur trioxide–pyridine complex served as the key sulfating reagent, allowing the introduction of sulfate groups onto hydroxyl or amino groups of the DK4 chain. Compared with protection-based semisynthesis [33], this approach avoids multiple protection and deprotection steps, while enzymatic and chemoenzymatic methods [34] generally offer greater positional selectivity but depend on specific sulfotransferases and an efficient PAPS supply system [35]. DK4-OS was prepared by reacting DK4 with the pyridine–sulfur trioxide complex (SO_3_·py) complex in anhydrous DMF at room temperature. For the synthesis of DK4-NS, DK4 was first deacetylated under alkaline NaOH conditions to expose free amino groups, followed by N-sulfation in the presence of Na_2_CO_3_ to maintain a suitable alkaline environment. For DK4-O,NS, DK4 was initially converted to its tetrabutylammonium salt by treatment with H^+^-form cation exchange resin and tetrabutylammonium hydroxide, and then dissolved in anhydrous DMF for sulfation. After precipitation, redissolution, and further sulfation, the desired O,N-sulfated derivative was obtained. All products were purified by precipitation, dialysis, concentration, and lyophilization to afford the corresponding sulfated DK4 derivatives. Thus, the present method was selected as an accessible strategy for comparative structure-activity studies rather than for producing fully sequence-defined natural structures. Because direct modification of an unprotected polymer may generate heterogeneous sulfate distributions and affect molecular weight or chain conformation, the products were characterized using NMR spectroscopic, elemental, and disaccharide composition analyses.

#### 2.1.1. NMR Spectra Analysis

**Figure 2:**
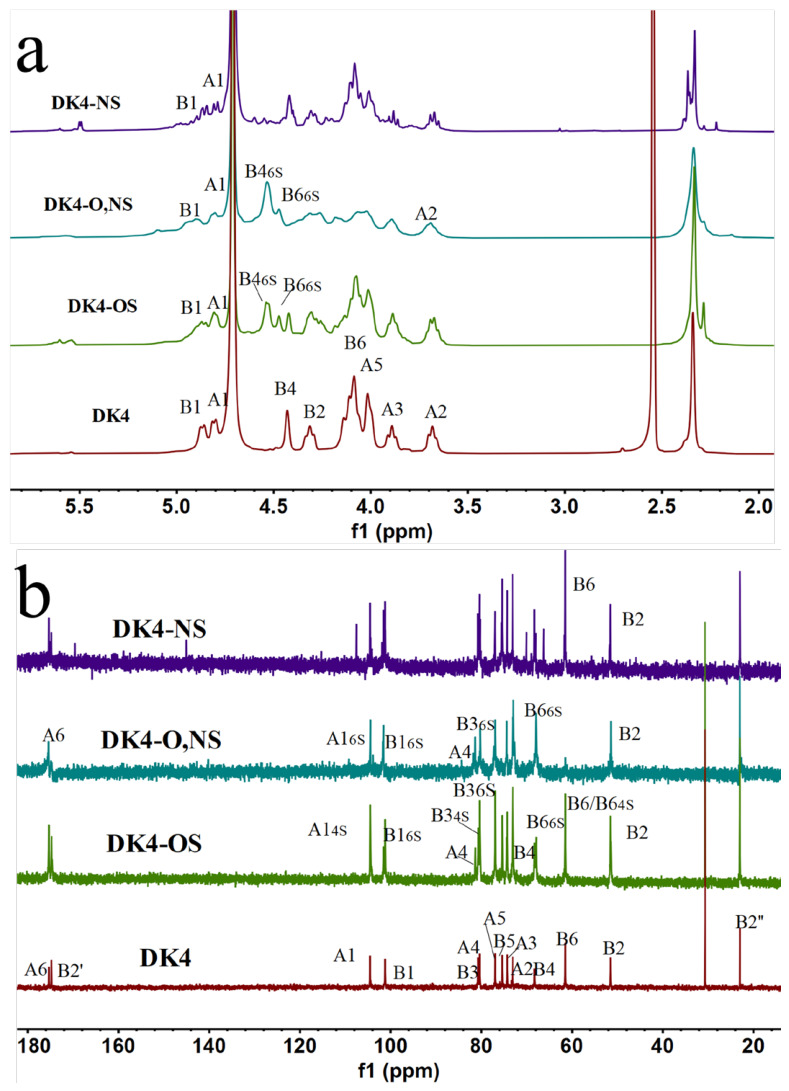
^1^HNMR spectra (**a**) and ^13^C NMR spectra (**b**) of DK4 and its derivatives, including DK4-OS, DK4-O,NS, and DK4-NS in D_2_O at 60 °C (from bottom to top). The ^1^H and ^13^C NMR spectra of DK4 replotted from the dataset reported previously [26].

The ^1^H and ^13^C NMR characteristics of unmodified DK4 have been reported in our previous study [26]. Therefore, only the principal signal assignments are briefly summarized here to provide a structural baseline for comparison with the sulfated DK4 derivatives. GlcA and GalNAc residues are marked as A and B, respectively. All samples displayed a characteristic pattern of proton signals located in the range of 3.50–5.20 ppm. The signals at approximately 2.32 ppm were assigned to acetamido methyl protons of GalNAc (Figure 2a). For DK4, ranges of 3.60–4.30 ppm were attributed to H2, H3, H5 of GlcA and H6 of GalNAc [32].

O-Sulfation generally alters the chemical environments of the protons at or near the substituted positions, with the corresponding resonances commonly appearing within the range of 3.90–4.80 ppm [36]. In the ^1^H NMR spectra of DK4-OS and DK4-O,NS, the signals at 4.48 and 4.53 ppm were assigned to B66S and B46S, respectively [26]. Meanwhile, the N-acetyl methyl proton signal at approximately 2.32 ppm remained clearly detectable in DK4-OS and DK4, indicating that the N-acetyl structures were largely retained after modification. In contrast, the N-acetyl methyl signal at approximately 2.32 ppm was markedly reduced in DK4-NS and DK4-O,NS.

In the spectrum of DK4, the signals at 4.80 and 4.85 ppm were assigned to H1 of GlcA and H1 of GalNAc, respectively. Following chemical modification, the B1 signal of DK4-O,NS shifted to 4.90 ppm, whereas smaller changes were observed for DK4-OS and DK4-NS, in which the B1 signals shifted to 4.87 and 4.86 ppm, respectively. These results suggest that the principal polysaccharide backbones of the three sulfated DK4 derivatives were largely preserved after chemical modification, while the observed chemical-shift changes reflect alterations in the local chemical environments of the sugar residues caused by sulfate group incorporation.

The structures were further verified by ^13^C NMR spectroscopy, as shown in Figure 2b. The signal at 23.0 ppm is related to the acetamido methyl carbon of the GalNA unit, marked as B2″. The signals at 175.3 and 174.7 ppm were related to the carbonyl carbons of GlcA (A6) and GalNAc (B2′) units, respectively. Compared with DK4, DK4 derivatives exhibited similar ^13^C NMR spectral profiles after sulfation, indicating that the chondroitin backbone was well preserved. The C-4 and C-6 positions of GalNAc were identified as the major sulfation sites [37]. For DK4-O,NS, the absence of the N-acetyl carbonyl carbon signal at approximately 174.7 ppm indicated successful deacetylation of the GalNAc residues.

Based on comparison with our previous NMR analyses of chondroitin sulfate A, C, and E [26], the peaks at 67.9 ppm in the spectrum of DK4-OS was tentatively assigned to the GalNAc6S (B66S). Sulfation of the GalNAc residues appeared to increase the heterogeneity of their local chemical environments, resulting in partial splitting or broadening of several carbon resonances. For DK4-OS, the split signals at 67.9 and 61.5 ppm corresponded to GalNAc6S (B66S) and GalNAc4S (B64S), respectively. The split signals at 80.4 and 80.7 ppm were consistent with GalNAc6S (B36S) and GalNAc4S (B34S), respectively. The peaks at 101.2 and 101.6 ppm were consistent with the anomeric C1 signals of GalNAc4S and GalNAc6S, respectively. The peak at 104.4 ppm was consistent with C1 of the GlcA residue (A14S).

Hence, based on the ^1^H and ^13^C NMR analyses, DK4-OS contained C4- and C6-sulfated GalNAc residues as the major components, whereas DK4-O,NS was mainly characterized by C6 O-sulfation together with partial N-sulfation of GalNAc. In DK4-NS, the weakened N-acetyl carbonyl carbon signal at approximately 174.7 ppm, compared with that of DK4, indicated partial deacetylation followed by N-sulfation, suggesting that only a fraction of the GalNAc residues was N-sulfated. Moreover, all three derivatives retained the characteristic carbon signals of the DK4 backbone, demonstrating that the polysaccharide skeleton was largely preserved during the derivatization process.

#### 2.1.2. Fourier Transform Infrared Spectroscopy (FTIR) Analysis

**Figure 3:**
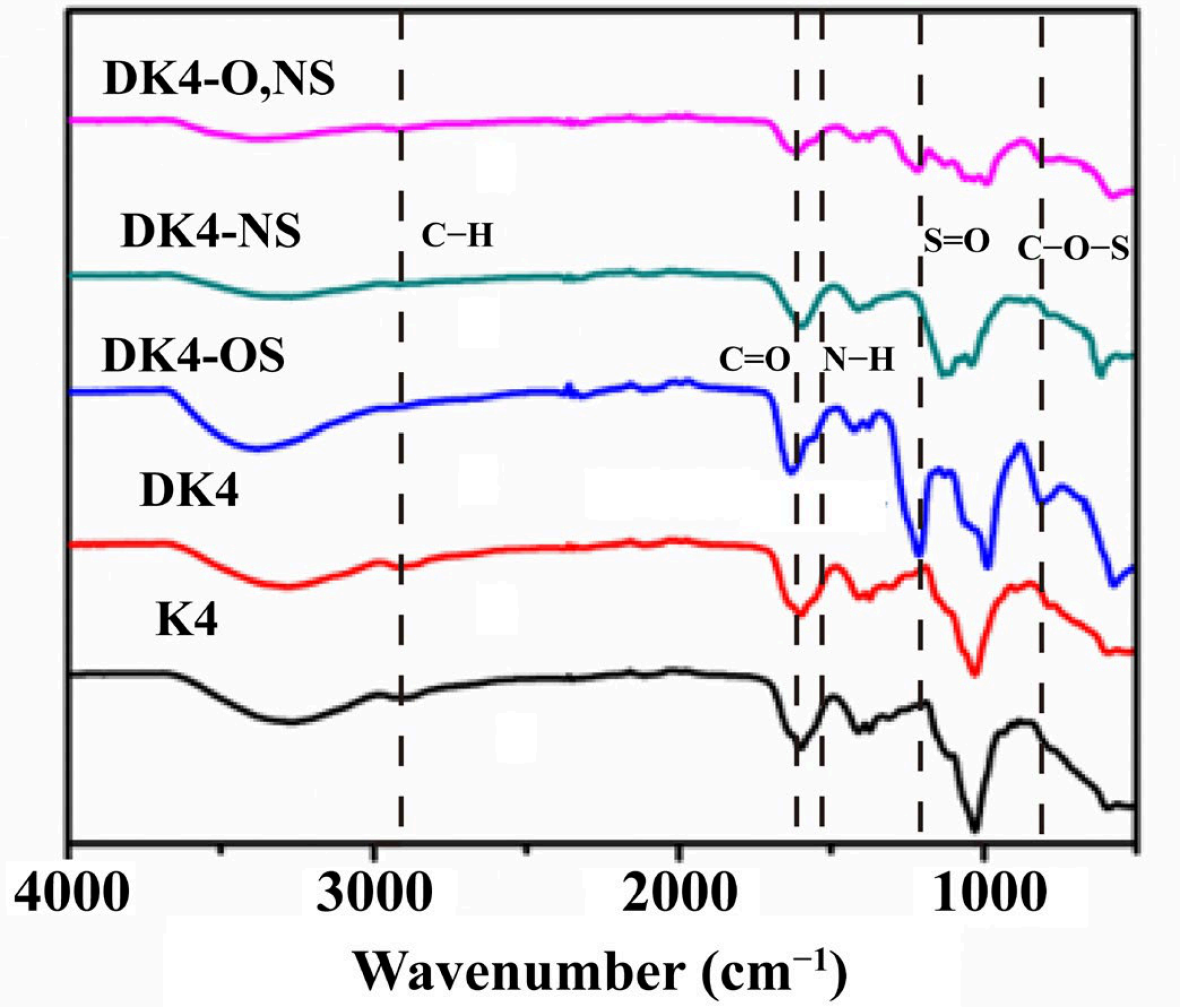
FTIR spectra of DK4 and its derivatives, including DK4-OS, DK4-O,NS, and DK4-NS (from bottom to top).

As shown in Figure 3, the FTIR spectra provided further evidence for the successful sulfation of DK4. The broad absorption band at 3200–3600 cm^−1^ was attributed to O−H stretching vibrations, while the band around 2910 cm^−1^ was assigned to asymmetric C−H stretching vibrations. The absorption bands at 1614 and 1540 cm^−1^ were ascribed to the −CO−NH group of GalNAc residues. The strong band at 1032 cm^−1^ was attributed to the skeletal vibrations of C−O and C−O−C bonds. Compared with DK4, DK4-OS, DK4-NS, and DK4-O,NS exhibited an obvious absorption band near 1240 cm^−1^ corresponding to the S=O stretching vibration of sulfate groups. In addition, the enhanced absorption bands in the 800–820 cm^−1^ region observed in the three derivatives could be assigned to C−O−S stretching vibrations. For DK4-NS and DK4-O,NS, the decreased intensity of the amide-related bands compared with DK4 suggested the formation of N-sulfated structures after deacetylation. Consequently, the FTIR, ^1^H NMR, and ^13^C NMR results demonstrated that O-sulfated, N-sulfated, and O,N-sulfated DK4 derivatives were successfully obtained through chemical modification, while the main polysaccharide backbone was largely preserved.

#### 2.1.3. Elemental Analysis

DK4 consists of repeating GlcA−GalNAc disaccharide units with a molecular formula of C_14_H_21_NO_11_, giving a theoretical N/C molar ratio of around 1:14 [26]. The elemental analysis results of the different chondroitin sulfate derivatives were shown in Table 1. The molar ratios of N/C in the four samples were around 1:14.43, 1:16.38, 1:14.67, and 1:14.94, respectively. The N/C ratios of DK4-OS (H), DK4-NS, and DK4-OS (L) were close to the theoretical value of the DK4 repeating unit. This result indicated that the GlcA−GalNAc backbone of DK4 was largely preserved after sulfation. The N/C ratio of DK4-O,NS was 1:16.38, which was slightly higher than the theoretical value of 1:14. This result suggested a relatively higher carbon content compared with nitrogen in this sample. This may be related to changes in GalNAc during N-deacetylation and N-sulfation.

Combined with the NMR analysis, the degree of sulfation (DS) values of DK4-OS (H) and DK4-O,NS were higher than 100%. Especially, DK4-OS (H) showed a DS value of 235.5%, indicating that dual sulfation of GalNAc residues was the major component. The DS value of DK4-O,NS was also higher than 100%, but lower than the level expected for complete disulfation. This result suggested that O,N-sulfated structures were likely the major components, while monosulfated or incompletely sulfated structures may also be present. DK4-NS showed a DS value of 58–61%, indicating that approximately 0.6 sulfate groups were introduced into each repeating disaccharide unit on average. Therefore, DK4-NS was considered a low-substitution sulfated product. Compared with DK4-OS (H), DK4-OS (L) showed a much lower DS value of 58–62%. These results indicated that the degree of O-sulfation could be effectively regulated by changing the reaction conditions.

#### 2.1.4. Disaccharide Analysis

The DK4 sulfated derivatives were digested with chondroitinase ABC (ChABC), and the digestion products were analyzed by HPLC at 232 nm [38]. ChABC specifically cleaves the β-(1→4) glycosidic linkage between GlcA and GalNAc residues. This reaction produces enzymatic fragments containing unsaturated uronic acid (ΔUA) at the non-reducing end. The unsaturated double bond of ΔUA shows a characteristic UV absorbance at 232 nm.

**Figure 4:**
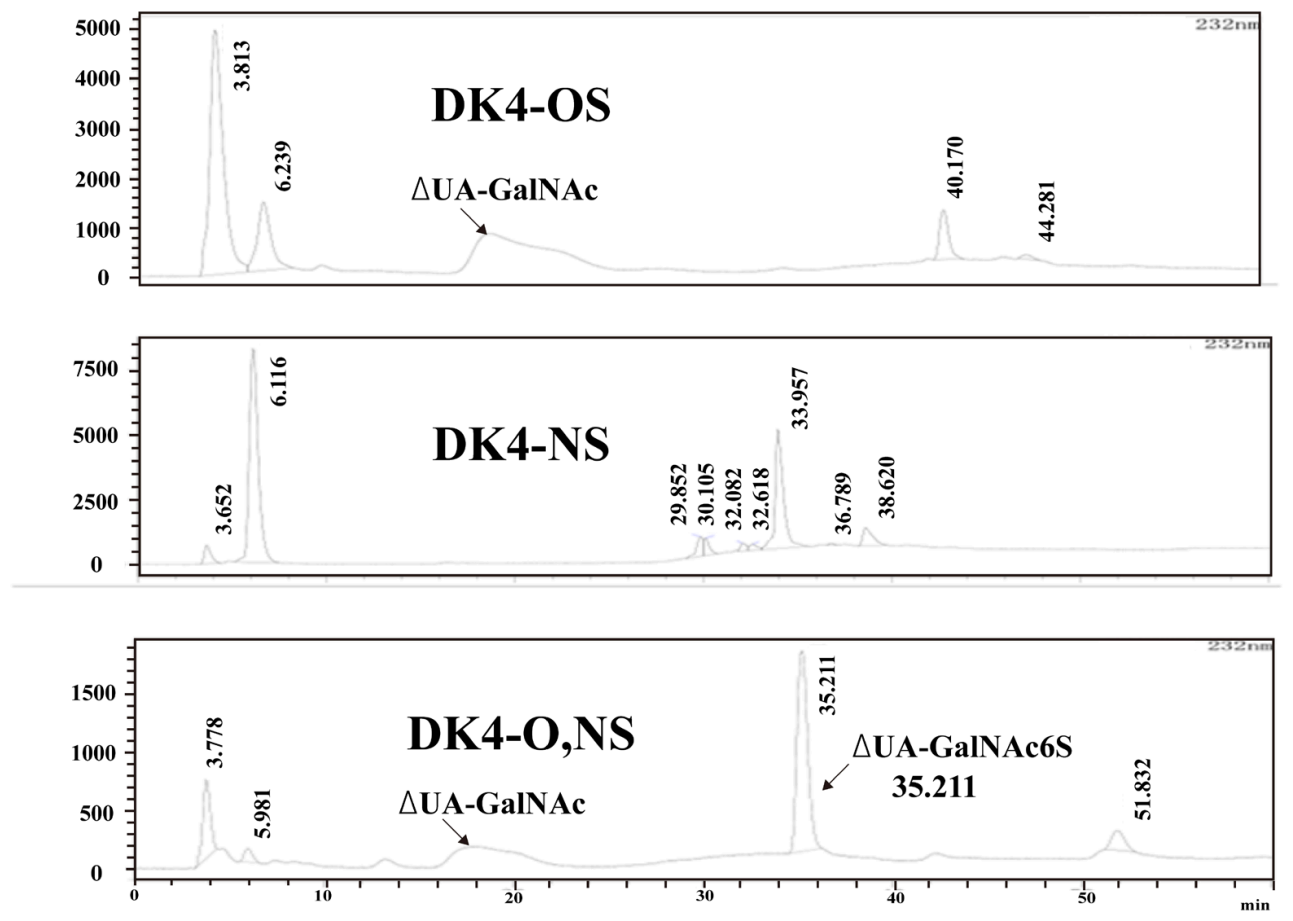
SAX-HPLC profiles of digested disaccharides from DK4-OS, DK4-O,NS, and DK4-NS.

The peaks eluted before 10 min were mainly attributed to salts or small molecular impurities from the digestion system, whereas the peak at 20 min was assigned to ΔUA-GalNAc [26,39]. In the chromatogram of DK4-OS, two peaks were observed at 40.170 and 44.281 min (Figure 4). The peak around 40 min was assigned to disulfated disaccharides. The later peak at 44.281 min may correspond to sulfated fragments with a higher O-sulfated derivative. Together with the elemental analysis results, these findings indicated that DK4-OS was a highly O-sulfated product. For DK4-NS, several small peaks appeared in the 29–39 min region, including signals at approximately 29.852, 30.105, 32.082, 32.618, 33.957, 36.789, and 38.620 min. The elution time near 35 min is generally associated with monosulfated disaccharides. Since DK4-NS was not a highly sulfated sample, ChABC digestion produced a certain amount of unsaturated sugar fragments, resulting in mixed peaks in this region. In the chromatogram of DK4-O,NS, the predominant elution peak observed at 35.211 min was attributed to ΔUA-GalNAc6S. A later elution peak was also observed at 51.862 min. These results suggested that DK4-O,NS contained monosulfated fragments as well as highly sulfated or O,N-sulfated fragments, which was consistent with the DS value.

In the present study, DK4-OS retained the chondroitin-like GlcA−GalNAc backbone and underwent chemical O-sulfation. DK4-OS may be considered to share certain sulfation features with CSE. However, it should not be classified as an authentic natural CS subtype because chemical sulfation may generate a broader and less sequence-controlled distribution of sulfate groups than enzymatic sulfation in natural CS biosynthesis. In contrast, DK4-NS was produced by de-N-acetylation followed by N-sulfation, resulting in noncanonical N-sulfated galactosamine (GalN) residues, whereas DK4-O,NS contained both O- and N-sulfated motifs [36]. Such N-sulfated galactosamine units are not characteristic components of natural CSA, CSC, or CSE, in which the galactosamine residue predominantly remains N-acetylated. DK4-NS and DK4-ONS should therefore be regarded as synthetic, noncanonical CS-like derivatives rather than counterparts of the classical natural CS subtypes.

Nevertheless, the present structural evidence, including ^1^H NMR and ^13^C NMR spectroscopy, supports the preservation of the major saccharide backbone in the DK4 derivatives. Elemental analysis further confirmed the successful introduction of sulfate groups, while disaccharide composition analysis provided preliminary information regarding their disaccharide structural features. In future studies, we plan to employ high-resolution mass spectrometry and tandem mass spectrometry to achieve a more comprehensive structural characterization. These approaches will allow more accurate determination of the chain-length distribution, substitution patterns, and specific modification sites of the DK4 derivatives.

### 2.2. Cell Assays

**Figure 5:**
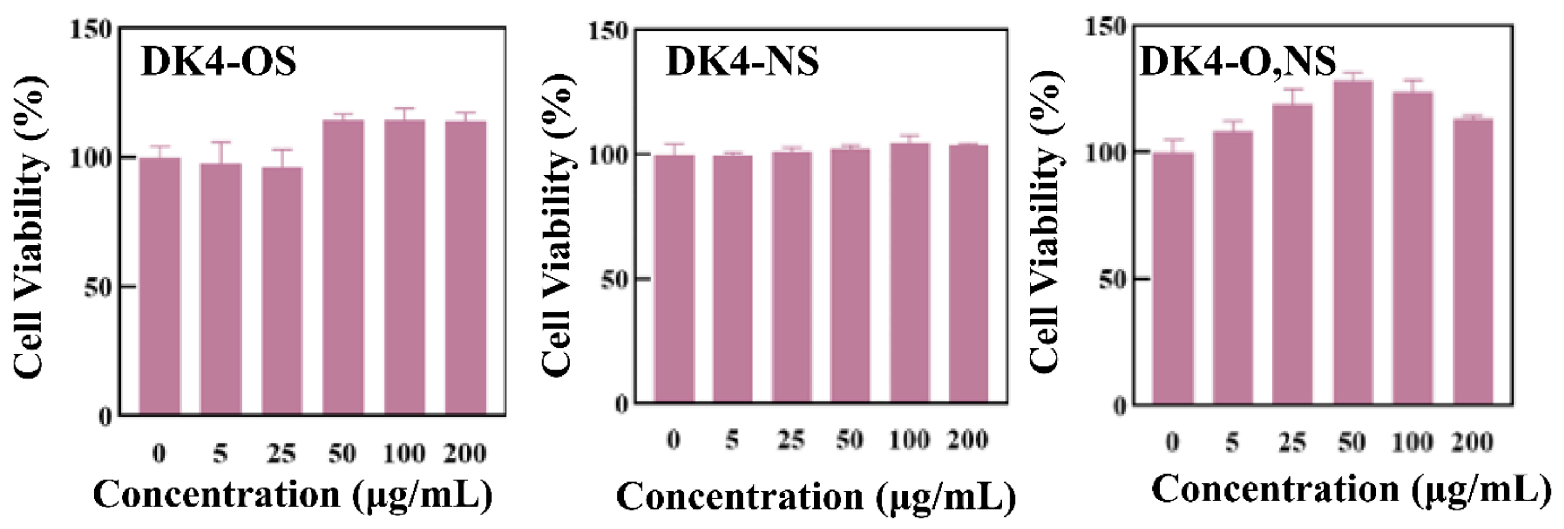
Effects of sulfated DK4 derivatives on the cell viability of AST/OPCs at 48 h.

The CCK-8 assay was used to evaluate the potential cytotoxicity of the DK4 derivatives toward astrocytes (AST) and oligodendrocyte progenitor cells (OPCs). The concentration range of 0–200 μg/mL was selected as an exploratory range, with reference to concentrations reported for chondroitin sulfate and related sulfated polysaccharides in cell-based assays [40,41]. As shown in Figure 5, the mixed cells were incubated with different concentrations of the derivatives for 48 h. None of the DK4 derivatives showed obvious cytotoxicity, and the cell viability remained above 90% in all treatment groups. Among these derivatives, DK4-OS increased the viability of AST/OPCs cells at concentrations of 50–200 μg/mL. These results indicated that the prepared DK4 derivatives were non-toxic to the cells. In addition, DK4-OS promoted cell proliferation within a certain concentration range.

**Figure 6:**
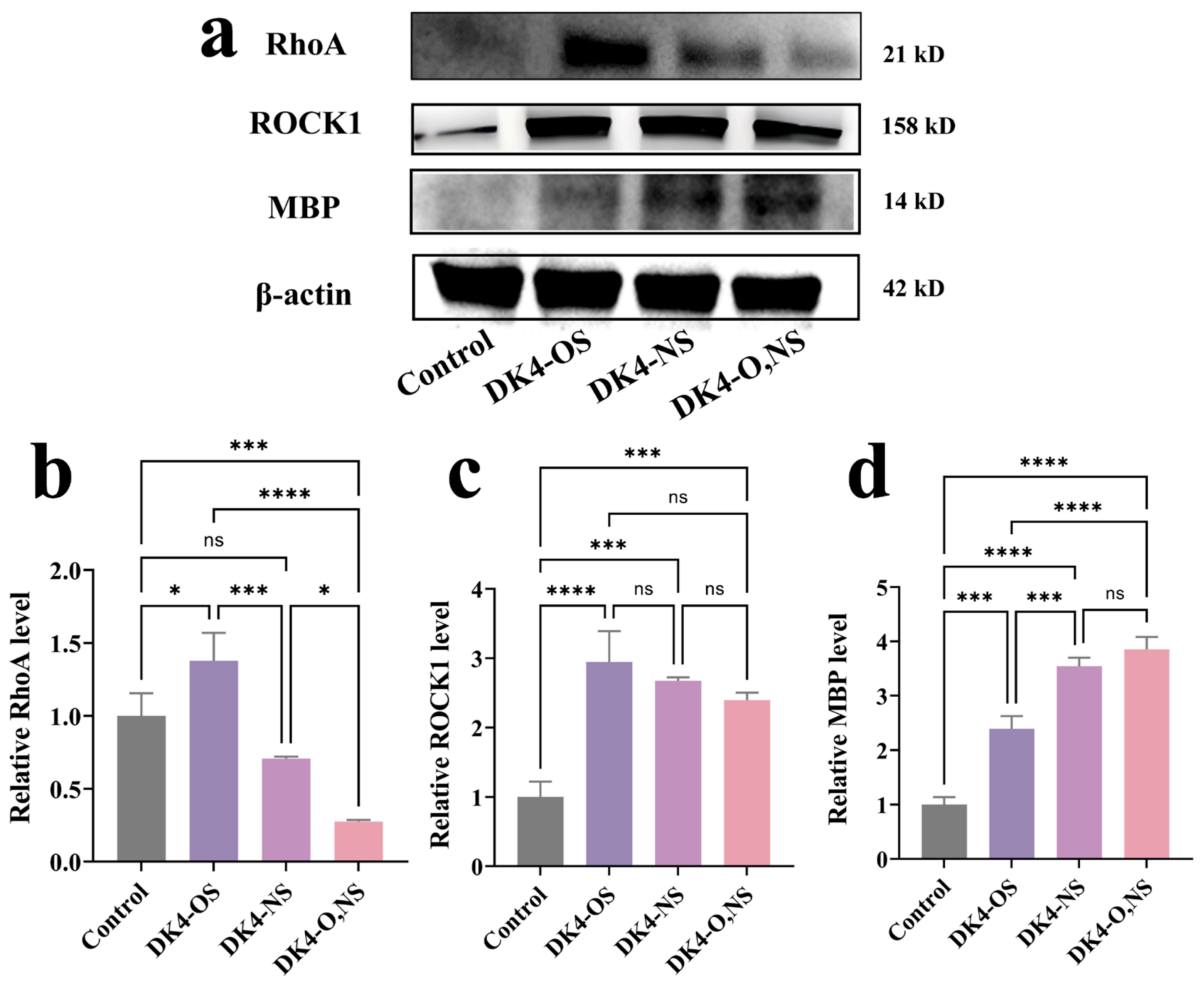
Western blotting analysis (**a**) in AST/OPCs stimulated by DK4-OS, DK4-NS, and DK4-O,NS for 48 h; their corresponding quantification plots of relative protein levels including RhoA (**b**), ROCK1 (**c**), MBP (**d**). (* *p* < 0.05, *** *p* < 0.001, **** *p* < 0.0001, “ns” means not significant).

The effects of DK4 derivatives on the expression of glial cell function-related proteins were further evaluated. Oligodendrocyte maturation and myelination are important processes in neural repair [42]. Myelin basic protein (MBP) is a key marker of oligodendrocyte maturation and myelin-related function [43]. In addition, the RhoA/ROCK1 signaling pathway is involved in cytoskeletal remodeling, cell morphology regulation, and process extension [44]. Therefore, Western blotting analysis was performed to detect the expression levels of ROCK1 and MBP. This was used to investigate the regulatory effects of DK4-OS, DK4-NS, and DK4-O,NS on oligodendrocyte maturation and related signaling pathways (Figure 6a).

Western blotting analysis showed that RhoA/ROCK1 expression was significantly increased after treatment with DK4-OS, DK4-NS, and DK4-O,NS compared with the Control group. The expression levels of RhoA in the DK4-OS, DK4-NS, and DK4-O,NS groups were approximately 1.38-, 0.71-, and 0.27-fold higher than the control group, respectively (Figure 6b). Notably, RhoA expression in the DK4-OS group was significantly higher than that in the DK4-NS and DK4-O,NS groups. These results suggest that different sulfation patterns confer distinct biological activities on DK4 derivatives. The expression levels of ROCK1 in the treated groups were approximately 2.9-, 2.6-, and 2.3-fold higher than the control group, respectively (Figure 6c). No significant differences were observed among the DK4 derivative groups. Meanwhile, all three derivatives significantly upregulated MBP expression. In particular, MBP expressions in the DK4-OS, DK4-NS and DK4-O,NS groups were approximately 2.4-, 3.5- and 3.8-fold higher than that in the control group, respectively (Figure 6d). MBP expression was significantly higher in the DK4-O,NS group than in the DK4-OS group, whereas no significant difference was detected between the DK4-O,NS and DK4-NS groups. The increased expression of MBP indicated that the sulfated DK4 derivatives may promote oligodendrocyte maturation and myelin-related protein expression. Collectively, the sulfated DK4 derivatives differentially altered the expression levels of RhoA/ROCK1, MBP.

## 3. Materials and Methods

### 3.1. Materials

Trifluoroacetic acid (TFA), SO_3_·py and tetrabutylammonium hydroxide were purchased from Aladdin (Shanghai, China). Na_2_CO_3_, acetone, *N*,*N*-dimethylformamide (DMF), Tween-20 and NaCl were purchased from Sinopharm Chemical Reagent Co., Ltd (Shanghai, China). Polylysine (PLL) and bovine serum albumin (BSA) were purchased from Solarbio Life (Beijing, China). The Cell Counting Kit-8 (CCK-8) assay kit was procured from Suzhou New Saimei Biotechnology Co., Ltd (Suzhou, China). Radioimmunoprecipitation assay (RIPA) lysis buffer was obtained from Beyotime Biotechnology (Shanghai, China). Penicillin-streptomycin (Pen/Strep), Dulbecco’s Modified Eagle’s Medium (DMEM), fetal bovine serum (FBS) were obtained from Gibco Invitrogen (Carlsbad, CA, USA). MBP Rabbit mAb, ROCK1 Rabbit mAb, RohA Rabbit mAb, and β-actin Rabbit mAb were obtained from Cell Signaling Technology (Beverly, MA, USA). All chemicals were used as received unless indicated otherwise. Deionized water was used in this work.

### 3.2. Sample Preparation

K4 polysaccharide was isolated and purified from cell cultures of engineered *Escherichia coli* K4 (ATCC 23502) as previously described [24]. K4 polysaccharide was hydrolyzed with 25 mM TFA in a sealed tube at 100 °C for 30 min, followed dialysis and lyophilization to obtain DK4.

#### 3.2.1. Synthesis of DK4-OS Derivative

DK4-OS was prepared using a modified SO_3_·py/DMF sulfation procedure based on previously reported methods [45]. DK4 (100 mg) was suspended in anhydrous DMF (4 mL) under stirring, followed by the addition of the SO_3_·py complex (340 mg) solution in DMF (1.5 mL). The reaction mixture was stirred at rt for 24 h. After completion, the product was precipitated with acetone saturated with NaCl (3.2 mL) and collected by low-speed centrifugation. The sediment was washed three times with saturated NaCl-acetone solution, dissolved in deionized water, neutralized with 1 M NaOH, dialyzed against water using a 1000 Da molecular weight cut-off dialysis bag (Solarbio Life Sciences Co., Ltd., Beijing, China), concentrated, and lyophilized.

#### 3.2.2. Synthesis of DK4-NS Derivative

An adapted method based on previously reported polysaccharide-modification procedures [46]. DK4 (100 mg) was suspended in 2 M NaOH solution (10 mL), heated to 60 °C, and stirred for 28 h. After cooling to rt, the reaction mixture was centrifuged, and the supernatant was adjusted to pH 7.0 with 2 M HCl. The resulting solution was heated to 40 °C, followed by the addition of Na_2_CO_3_ (160 mg), and SO_3_·py complex (260 mg) was added portionwise over 4 h. After cooling to rt, the solution was adjusted to pH 7.4 with 1 M NaOH, dialyzed against water using a 1000 Da molecular weight cut-off dialysis bag, concentrated, and lyophilized.

#### 3.2.3. Synthesis of DK4-O,NS Derivative

An adapted method based on previously reported polysaccharide-modification procedures [47]. DK4 (100 mg) was dissolved in water (20 mL) and fully exchanged using H^+^-form cation-exchange resin, followed by lyophilization. The obtained product was dissolved in deionized water (10 mL), neutralized with tetrabutylammonium hydroxide solution, and lyophilized to yield the product (70 mg). The product was dissolved in anhydrous DMF (3.5 mL), followed by the portionwise addition of SO_3_·py complex (300 mg). The reaction mixture was heated to 50 °C and stirred for 24 h. The mixture was heated to rt and precipitated with acetone saturated with NaCl. The sediment was dissolved in 0.2 M NaCl solution (1 mL), adjusted to pH 7.8 with 2 M NaOH, and precipitated again with acetone. The sediment was dissolved in water (2 mL), heated to 40 °C, followed by the addition of Na_2_CO_3_ (112 mg), and the SO_3_·py complex (112 mg) was added in 4 portions over 4 h. The reaction was continued for another 24 h, followed by dialysis and lyophilization.

### 3.3. Characterization

Fourier transform infrared (FTIR) spectroscopy was performed using a NEXUS (Thermo Nicolet, Madison, WI, USA). ^1^H and ^13^C nuclear magnetic resonance (NMR) experiments were performed on a Bruker Advance III 400 MHz spectrometer (Bruker BioSpin, Billerica, MA, USA) at 60 °C. The polysaccharides were dissolved in D_2_O at concentrations of 25 mg/mL. Elemental analysis of polysaccharide derivatives was performed by using a Vario Micro Cube (Elementar, Langenselbold, Germany). The cytotoxicity assay was performed to attain the absorbance of samples in 96-well plates at 450 nm using a SpectraMax M2 microplate reader (Molecular Devices, Sunnyvale, CA, USA).

### 3.4. Disaccharide Analysis

Disaccharide analysis of the synthetic CS derivatives was performed as previously described [26]. Briefly, the samples were dissolved in buffer and digested with chondroitinase ABC (ChABC) at 37 °C for 24 h. The reaction was terminated by boiling, followed by centrifugation. The supernatant was desalted using a Sephadex G-25 M column and analyzed using HPLC (Shimadzu, Japan).

### 3.5. Cell Culture

AST and OPCs were isolated and cultured according to a previously reported method [8]. Briefly, cerebral cortices were collected from postnatal 24 h Sprague–Dawley (SD) rats and gently placed in cold PBS, which were digested with 0.125% trypsin for 10 min. The cell suspension was then centrifuged, and the resulting cell pellet was resuspended in DMEM. The cell suspension was incubated at 37 °C for 30 min to reduce fibrocytes. The supernatant was transferred into T75 flasks precoated with PLL (0.01%), and the cells were cultured in complete medium composed of 89% DMEM, 10% FBS, and 1% Pen/Strep. After approximately 10 days of culture, the mixed glial cells were shaken for 1 h (37 °C, 230 rpm) to remove microglia. The remaining adherent cells were then maintained in proliferation medium to give AST/OPCs coculture.

### 3.6. CCK-8 Assay

The cytotoxicity of the materials was evaluated using CCK-8. AST/OPCs (1 × 10^5^/mL) were seeded into 96-well plates and incubated at 37 °C with 5% CO_2_ to allow attachment. Then, the cells were treated with varying concentrations (0, 5, 25, 50, 100, and 200 μg/mL) of DK4-OS, DK4-O,NS, and DK4-NS for 48 h at 37 °C in a 5% CO_2_-humidified atmosphere. Subsequently, 10 μL of CCK-8 solution was added to each well, followed by further incubation for 1.5 h. The optical density (OD) was then measured at 450 nm using a SpectraMax M2 microplate reader. 

### 3.7. Western Blotting Analysis

AST/OPC cells were cultured under the same conditions and treated with the indicated concentrations of DK4 fractions for 48 h. After treatment, cells were lysed in RIPA buffer supplemented with protease and phosphatase inhibitors for 15 min. Protein concentrations were quantified using BSA as a standard. Equal amounts of protein (30 μg per lane) were separated by 8% sodium dodecyl sulfate-polyacrylamide gel electrophoresis (SDS-PAGE) and transferred onto PVDF membranes (PerkinElmer, Waltham, MA, USA). The membranes were blocked with TBST buffer (5% fat-free milk in PBS containing 0.1% Tween-20) for 1 h at room temperature and then incubated overnight at 4 °C with primary antibodies against target proteins, followed by incubation with horseradish peroxidase (HRP)-conjugated secondary antibodies for 1 h at room temperature. After five washes with TBST, protein bands were visualized using an enhanced chemiluminescence (ECL) substrate and imaged using a Tanon 5200 Multi Chemiluminescence Imaging System (Tanon, Shanghai, China) [48,49].

### 3.8. Statistical Analysis

One-way ANOVA and SPSS v.16.0 software were used for the statistical analysis. Quantification data are presented as the mean ± standard deviation from triplicate trials. *p* values less than 0.05 were used as the threshold for a statistically significant difference (* *p* < 0.05, ** *p* < 0.01, *** *p* < 0.001, **** *p* < 0.0001, “ns” means not significant).

## 4. Conclusions

Three sulfated derivatives were semisynthesized through chemical modification of the DK4 polysaccharide produced by engineered *Escherichia coli* K4. Their chemical structures and sulfation degrees were systematically characterized by ^1^H NMR, ^13^C NMR, HPLC, and elemental analysis. The DS values of DK4-OS, DK4-O,NS, and DK4-NS were 242.9%, 184.7%, and 60.6%, respectively, indicating that DK4-OS had the highest sulfation degree, followed by DK4-O,NS, whereas DK4-NS showed the lowest sulfation degree. The CCK-8 assay showed that the sulfated DK4 derivatives exhibited no cytotoxicity toward AST/OPCs cocultures at concentrations ranging from 0 to 200 μg/mL, suggesting good biocompatibility and providing a basis for further biological evaluation. Western blotting analysis indicated that the DK4 derivatives altered the expression of RhoA, ROCK1, and MBP. Among them, DK4-NS and DK4-O,NS showed more pronounced effects in promoting MBP expression. These findings indicate that the biological effects of the DK4 derivatives were not determined solely by their total sulfur content or overall degree of sulfation. Instead, the position of the sulfate groups and the accompanying changes in the N-acetylation state jointly contribute to their biological activities. Overall, this study provides experimental evidence that sulfation pattern, rather than sulfation degree alone, is an important structural factor affecting the biological functions of DK4 derivatives and lays a foundation for the further development of chondroitin sulfate analogues for studies of oligodendrocyte regulation and nerve repair.

## Figures and Tables

**Table 1:** Elemental analysis of sulfated DK4 derivatives.

Sample	Nitrogen	Carbon	Hydrogen	Sulfur	DS ^1^ %	DS ^2^ %
DK4-OS (H)	1.78	22.03	4.468	9.884	235.5	242.9
DK4-O,NS	1.89	26.55	4.755	7.981	157.8	184.7
DK4-NS	2.56	32.21	5.303	3.549	57.8	60.6
DK4-OS (L)	2.35	30.11	4.902	3.323	57.9	61.9

^1^ Degree of substitution, DS = 5.25 × (S%/C%). ^2^ Degree of substitution, DS = 0.4375 × (S%/N%).

## Data Availability

The original contributions presented in this study are included in the article. Further inquiries can be directed to the corresponding author.
